# Molecular and cellular function of the proprotein convertase subtilisin/kexin type 9 (PCSK9)

**DOI:** 10.1007/s00395-015-0463-z

**Published:** 2015-01-20

**Authors:** Rainer Schulz, Klaus-Dieter Schlüter, Ulrich Laufs

**Affiliations:** 1 Physiologisches Institut, Justus-Liebig Universität Giessen, Aulweg 129, 35392 Giessen, Germany; 2 Klinik für Innere Medizin III, Kardiologie, Angiologie und Internistische Intensivmedizin, Universitätsklinikum des Saarlandes, 66421 Homburg/Saar, Germany

**Keywords:** PCSK9, Heart, Cardiovascular disease, Cholesterol

## Abstract

The proprotein convertase subtilisin/kexin type 9 (PCSK9) has emerged as a promising treatment target to lower serum cholesterol, a major risk factor of cardiovascular diseases. Gain-of-function mutations of PCSK9 are associated with hypercholesterolemia and increased risk of cardiovascular events. Conversely, loss-of-function mutations cause low-plasma LDL-C levels and a reduction of cardiovascular risk without known unwanted effects on individual health. Experimental studies have revealed that PCSK9 reduces the hepatic uptake of LDL-C by increasing the endosomal and lysosomal degradation of LDL receptors (LDLR). A number of clinical studies have demonstrated that inhibition of PCSK9 alone and in addition to statins potently reduces serum LDL-C concentrations. This review summarizes the current data on the regulation of PCSK9, its molecular function in lipid homeostasis and the emerging evidence on the extra-hepatic effects of PCSK9.

## Protein convertase–LDL receptor interaction

The main function of the proprotein convertase subtilisin/kexin (PCSK) type 9 (PCSK9) is the proteolytic maturation of secreted proteins such as hormones, cytokines, growth factors, and cell surface receptors [149]. The name PCSK9 stems from the relation to bacterial subtilisin and yeast kexin and the presence of nine secretory serine proteases. PCSK9 is expressed mainly in the liver, the intestine, the kidney, and the central nervous system [122].

**Fig. 1:**
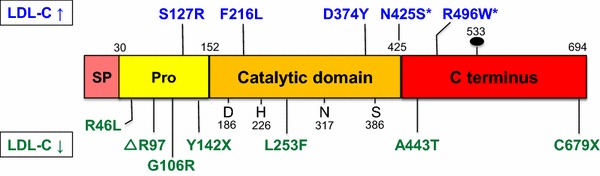
The structure of PCSK9 contains a signal peptide (SP, amino acids 1–30), a prodomain (Pro, amino acids 31–152), a catalytic domain and the C-terminal domain. The cleavage of the prodomain is required for PCSK9 folding and maturation. The location of the aspartic acid (D), histidine (H) and serine (S) comprising the catalytic triad and the site of binding of the single N-linked sugar (Asn533) are shown. The oxyanion hole is located at Asn317. Mutations associated with elevated plasma levels of LDL-C are depicted at the top (*blue*), mutations leading to reduced LDL-C at the bottom (*green*). The *asterisk* indicates mutations associated with elevated plasma LDL-C levels found only in families who also have mutations in the LDL receptor (modified from [75])

PCSK9 is a 692 amino acid protein with a molecular weight of 72 kDa that consists of a prodomain (PD), a catalytic domain and a cysteine- and histidine-rich C-terminal domain (CHRD) [41] (Fig. 1). The best characterized function of PCSK9 relates to the binding to LDL-C receptors (LDLR) in hepatocytes. Pro-PCSK9 (72 kDa) is synthesized in the endoplasmic reticulum (ER) as is the precursor form (120 kDa) of the low-density lipoprotein (LDL) receptor (LDLR). The binding of pro-PCSK9 to the LDLR in the ER supports the transport of the LDLR from the ER [163] towards the Golgi complex, where the LDLR acquires its mature carbohydrate residues (160 kDa). Trafficking of pro-PCSK9 to the Golgi apparatus depends on the presence of the protein Sec24A [31]. Within the Golgi, the pro-domain of pro-PCSK9 is auto-catalytically cleaved off, but remains non-covalently bound to the mature PCSK9 assisting the folding of PCSK9, and blocking its catalytic activity [61]. Binding of pro-PCSK9 to the precursor form of the LDLR promotes PCSK9 auto-catalytic cleavage [163].

Some of the loss-of-function (LOF) mutations of PCSK9—as the exchange of amino acids C678X [15] or S462P [25]—abolish the release of PCSK9 from the ER as does loss of parts of its PD [49]. On its way through the Golgi and trans-Golgi complex, PCSK9 co-localizes with the protein sortilin; in sortilin-knockout mice the plasma PCSK9 concentration is decreased suggesting that such protein–protein interaction is required for cellular secretion of PCSK9 [66]. In healthy humans, circulating PCSK9 levels directly correlate with plasma sortilin levels [66]. The exchange of amino acids S127R and D124G reduces secretion of PCSK9 from hepatocytes and increases the intracellular expression of PCSK9 [72]. It appears that partial proteolysis of PCSK9 is required prior to its cellular secretion [36]. Proteolysis of PCSK9 is regulated by phosphorylation at its residues serine 47 (PD) and serine 688 (CHRD) which occurs by a Golgi casein kinase-like kinase; an increase in epitope phosphorylation reduces proteolysis of PCSK9 [45].

Apart from acting as a chaperone to transport the precursor form of the LDLR from the ER, intracellular PCSK9 plays a role in regulating the expression of the mature LDLR by inducing intracellular degradation of the LDLR prior to its transport to the cell surface membrane. Given the fact that the mature LDLR and PCSK9 are found in the Golgi complex, it is likely that the LDLR degrading effect of PCSK9 occurs in or is initiated in the Golgi or trans-Golgi complex [107, 108]. The post-ER mechanism of LDLR degradation requires the catalytic activity of PCSK9 [13, 14].

If not degraded intracellularly, the mature LDLR is transported to the cell surface, where it resides in clathrin-coated pits because of its interaction with the low-density lipoprotein receptor adapter protein 1, which may cause autosomal recessive hypercholesterolemia (ARH). The LDLR undergoes endocytosis in the presence or absence of its ligand, entering the endocytic recycling compartment. The change in pH within this compartment allows dissociation of the LDLR from its ligand, which then becomes degraded in the lysosome while the LDLR recycles.

**Fig. 2:**
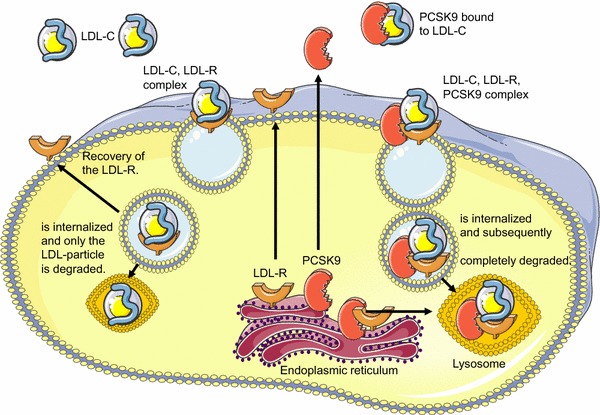
Schematic overview about the cellular regulation of PCSK9 and LDLR expression

The main role of secreted extracellular PCSK9 is to post-translationally regulate the number of cell surface LDLR. Secreted PCSK9 binds to the epidermal growth factor repeat A (EGF-A) region of the LDLR [21, 32, 179]. For such binding, the catalytic activity of PCSK9 is not required [101, 115], but pH changes and changes in the positive [70] or negative [71] charges of PCSK9 epitopes affect its binding affinity to the LDLR [16, 62]. Mutations in the EGF-A binding domain of the LDLR associated with familiar hypercholesterolemia increases PCSK9 binding [114]. The formed PCSK9–LDLR complex is internalized again by clathrin-mediated endocytosis [124, 130] and the complex is then routed to the sorting endosome/lysosome via a mechanism that does not require ubiquitination [172], but might involve interaction of the cytosolic tail of PCSK9 with the amyloid precursor protein like protein 2 [44]. At the acidic pH of the endosome/lysosome, an additional interaction between the ligand-binding domain of the LDLR and the C-terminal domain of PCSK9 occurs [49, 142]; as a consequence PCSK9 remains bound to the LDLR and the LDLR fails to adopt a closed conformation which is required for LDLR recycling. The failure of the LDLR to recycle appears to also involve ectodomain cleavage by a cysteine cathepsin in the sorting endosome [97]. Thus, by binding to the LDLR, PCSK9 disrupts the recycling of the LDLR leading to its degradation and subsequently a reduced number of available LDLRs. LDLR lacking its cytoplasmic domain are also degraded by PCSK9 [162] (Fig. 2).

PCSK9 undergoes self-assembly and forms PCSK9 dimers or trimers which have greater LDLR degrading activity [53]. One of the gain-of-function (GOF) mutations of PCSK9 (D374Y) is characterized by an enhanced PCSK9 self-assembly [53]. The main route of PCSK9 elimination is through LDLR binding [167], although LDLR-independent mechanisms of PCSK9 clearance must exist [24]. Up to 30 % of PCSK9 is bound to LDL-C in mice [55, 167] and normolipidemic subjects [84]. In mice, PCSK9 is also bound to high-density lipoprotein (HDL) [55]. For the binding of PCSK9 to LDL-C the amino residues 31–52 of the PD are required [84].

PCSK9 is cleaved by furin as well as protein convertases (PC) 5/6 [15] between the amino acids Arg 218 and Gln 219 [52], and both forms of PCSK9 can be measured in human plasma [67]. Furin-cleaved PCSK9 (55 kDa) is still active and binds to the LDLR, however, with a twofold reduced activity [104]. Indeed, injection of furin-cleaved PCSK9 into mice results in increased LDL-C, as LDLR are downregulated [104].

PCSK9 binds to a variety of other proteins (for review, see [178]), one of them being annexin A2 which is present in the nucleus, the cytosol and the cell membrane in a variety of cells. The N-terminal repeat R1 of annexin 2 binds to the CHRD region of PCSK9 and inhibits its extracellular LDLR degrading activity [109]. In annexin A2 knockout mice plasma PCSK9 levels are doubled resulting in reduced LDLR expression and an increase in LDL-C [151]; thus annexin A2 is viewed as endogenous inhibitor of PCSK9 [109].

In summary, PCSK9 regulates the concentration of circulating low-density lipoproteins by enhancing the degradation of the hepatic LDLR that is required for hepatic LDL-C clearance.

## Plasma concentration of PCSK9

The plasma concentration of PCSK9 follows a diurnal rhythm similar to cholesterol synthesis [28], with an increased plasma concentration in the morning and a lower concentration in the afternoon [125]. The plasma PCSK9 concentration is higher in women compared to men [90], and the PCSK9 concentrations decrease with age in men, but increase in women [11], most likely because elevated estrogen levels reduce PCSK9 expression [126]. Plasma PCSK9 concentration varies over a 50 [34]–100 [90] fold range [30–3,000 ng/ml] and plasma PCSK9 concentration correlates to plasma LDL-C concentration [4, 90] even in newborn infants [6]; in adults a 100 ng/ml increase in the plasma PCSK9 concentration will increase LDL-C by 0.20–0.25 mmol/l [92]. Lipid apheresis reduces plasma PCSK9 levels by 50 % [168], removing both the mature and the furin-cleaved form of PCSK9 [74].

## Regulation of PCSK9 gene expression

**Fig. 3:**
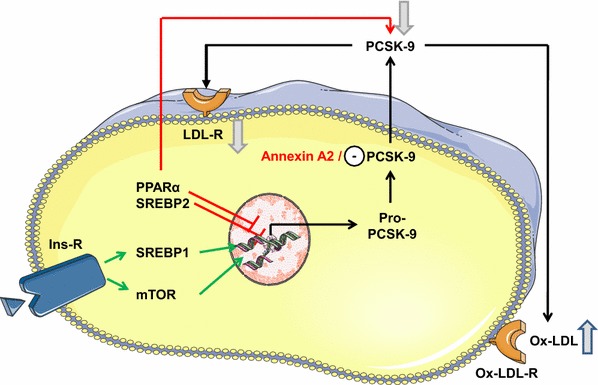
Activation of PCSK9 expression can be mediated by activation of insulin receptors (Ins-R) and subsequent activation of the sterol-response element binding protein (SREBP) 1 and mammalian target of rapamycin (mTOR) pathways. Reduction of PCSK9 expression can be achieved by peroxisome proliferator-activated receptor alpha (PPARα) and activation of SREBP2. Secretion of PCSK9 can be attenuated by annexin A2. Plasma concentration of PCSK9 can be reduced by PPARα-dependent cleavage (requiring furin). High concentrations of PCSK9 down-regulate LDLR expression and favor the formation of oxidized (ox)-LDL. See text for more details

A number of transcription factors or cofactors regulate the PCSK9 gene expression (Fig. 3), including sterol-response element binding proteins (SREBP-1/2). Since the PCSK9 gene is regulated by sterols through SREBP2, low dietary cholesterol concentrations potently suppresses its expression and PCSK9 protein levels decrease in the course of fasting and increase after feeding in animals [175] and humans [23]. SREBP2 also controls LDLR expression.

SREBP1 expression in hepatocytes is increased by insulin resulting in increased PCSK9 expression [39]. However, insulin can also activate the mammalian target of rapamycin complex 1 (mTORC1)/protein kinase δ pathway thereby inhibiting hepatocyte nuclear factor 1α (HNF1α) resulting in less PCSK9 expression in hepatocytes [2]. Indeed, in HepG2 cells, hyperinsulinemia decreases PCSK9 expression, an effect which is also observed in post-menopausal obese women [9]. On the contrary, in healthy men 24 h hyperinsulinemia did not alter plasma PCSK9 concentrations [80] and PCSK9 expression is similar in normal, pre- and Typ2-diabetic patients [22]. Thus, the overall effect of insulin on PCSK9 expression appears to be neutral.

Peroxisome proliferator-activated receptor (PPAR) regulates PCSK9 expression: PPARα reduces PCSK9 promoter activity thereby attenuating PCSK9 expression, while at the same time PPARα enhances furin/PC5/6 expression leading to increased cleavage of PCSK9 [85]. On the contrary, PPARγ increases PCSK9 expression in hepatocytes [50].

Other transcription factors or factors are the farnesoid X receptor (FXR, activated by bile acids, reduces PCSK9 expression) [93], the liver X receptor (LXR, activated by oxysterols, increases PCSK9 expression) [39, 148], and histone nuclear factor P (HINFP, increases PCSK9 expression) [100].

Also sirtuins 1 and 6 (SIRT1/6), critical histone deacetylases, suppress the PCSK9 gene [166], reduce PCSK9 secretion and increase hepatocyte LDLR expression [117] thereby modifying LDL-C homeostasis. Finally, the adipose tissue-derived adipokine resistin increases the PCSK9 expression and reduces LDLR expression in hepatocytes [116].

Ongoing studies will be instrumental for the understanding of the importance of the regulators of PCSK9 gene expression for serum LDL-C concentrations. In addition, further research is needed to address the expression of PCSK9 and its function in different compartments (e.g. blood, liver and intestine).

## PCSK9 and inducible degrader of LDLR (IDOL)

IDOL is another protein which involved in the internalization and degradation of the LDLR [77, 144]. IDOL binds to the C-terminus of the LDLR [147, 148] and stimulates clathrin-independent endocytosis of the LDLR [147]. IDOL, which is activated through the LXR employs the endosomal sorting complex required for transport to traffic LDLR to the lysosomes [147]. IDOL can also stimulate SREBP2 thereby increasing PCSK9 expression again reducing LDLR expression. Individuals carrying an IDOL mutation (pArg266X) which results in a complete loss of IDOL function exhibit low serum LDL-C concentrations [156].

## Drug-induced changes in PCSK9 expression

Given the number of transcription factors and co-factors regulating the PCSK9 gene it appears obvious that a number of drugs will affect PCSK9 expression (Table 1).

**Table 1:** Drugs affecting PCSK9 expression

	Direct (TF)	Indirect (LDL)	PCSK9 Expression	Transcription factor (TF) involved
Statins	+	+	Increased	SREBP2; HNF1α
Fibrates	+	+	Direct effect: reduced;	PPARα
			Indirect effect: increased	SREBP2
Ezetimibe	–	+	Indirect effect: increased	SREBP2
Insulin	+	–	Increased	SREBP1
			Reduced	HNF1α
Glitazones	+	–	Reduced	PPARγ
Rapamycine	+	–	Reduced	HNF1α
Berberine	+	–	Reduced	HNF1α
Resistin	na	na	Reduced	na

*Direct* direct effect through modulation of TF, *indirect* indirect through reduction of LDL-cholesterol and subsequent activation of TF, *SREBP* sterol-response element binding protein, *HNF1α* hepatocyte nuclear factor 1α, *PPAR* peroxisome proliferator-activated receptor, *na* not assessed

*Statins* increase the transcription factor SREBP2 [7] thereby increasing PCSK9 expression [7, 18, 138] dose-dependently [64] also in diabetic patients (otherwise having normal PCSK9 levels, see above) [29, 40, 120]. More recently, statins have been shown to increase HNF1α expression in hepatocytes, thereby increasing PCSK9 expression to a greater extent than LDLR expression [48]. Statins not only enhance the monomeric but also the heterodimeric form of PCSK9 [123]. The increase in PCSK9 expression following statin treatment is correlated to the statin-induced LDL-C decrease [10], and can be reversed by mevalonate treatment [51] or resistin treatment [116]. Such co-treatment therefore could enhance the LDL-C reducing effect of statins.

*Fibrates* activate PPARα thereby affecting PCSK9 expression [85]. Indeed, fibrates reduce PCSK9 expression in hepatocytes [110] and in patients [91]. However, the latter finding is controversial since fibrate treatment increased PCSK9 in another short-term patient study [169]; this discrepant finding can potentially be explained by the LDL-C lowering effect of fibrates leading to an increased PCSK9 expression (for review, see [12]).

*Ezetimibe* does not increase PCSK9 *per se* in healthy men [18]. However, ezetimibe through its plasma LDL-C concentration reducing effect might lead to a secondary increase of PCSK9 expression as measured in cynomolgus monkeys [68].

*Cholesterylester transfer protein (CETP) inhibitors*, in contrast downregulate PCSK9 and LDLR expression through decreases in SREBP2 expression in hepatocytes [47].

*Glitazones* activate the extracellular-regulated kinases (ERK) 1 and 2 resulting in phosphorylation of PPARγ thereby reducing its activity; as PPARγ increases PCSK9 mRNA and protein expression in the liver, glitazones attenuate secretion of PCSK9 from hepatocytes [50].

*Rapamycin*, as an immunosuppressant, attenuates mTORC1 activation thereby increasing HNF1α activity and subsequently PCSK9 expression [2].

*Berberine* treatment decreases PCSK9 in hepatocytes associated with an inhibition of the transcription factor HNF1α [26, 99]. Interestingly, in in vivo studies, berberine treatment reduces dyslipidemia induced by LPS treatment which was associated with a reduction in the plasma PCSK9 concentration [177].

## Disease-induced changes of PCSK9 expression

Inflammation stimulates PCSK9 expression in hepatocytes [54] and the plasma PCSK9 concentration is correlated to white blood cell count [102] and fibrinogen concentration [181] in patients. In patients with an acute myocardial infarction, the plasma PCSK9 concentration is elevated compared to stable coronary artery disease patients [5]. Similarly in proteinuric patients [87] and those with a nephrotic syndrome [79] an increase in plasma PCSK9 concentration occurs; part of this effect, however, might relate to the statin treatment of these patients.

## PCSK9 mutations and LDL-C concentration

Alterations in the PCSK9 gene and/or PCSK9 GOF mutations are responsible in part for familiar hypercholesterolemia (FH) [106], including the autosomal dominant form. GOF mutations of PCSK9 [1, 15, 72] lead to hypercholesterolemia while non-sense mutations [1] or LOF-mutations [15, 17, 34, 45] reduce the LDL-C concentration (Fig. 1). GOF mutations are sometimes related to reduced furin cleavage of PCSK9 [52] while LOF-mutations relate to lack of PCSK9 phosphorylation and subsequent increased proteolysis [45]. For review, please refer to [170].

## PCSK9 and cardiovascular disease

**Fig. 4:**
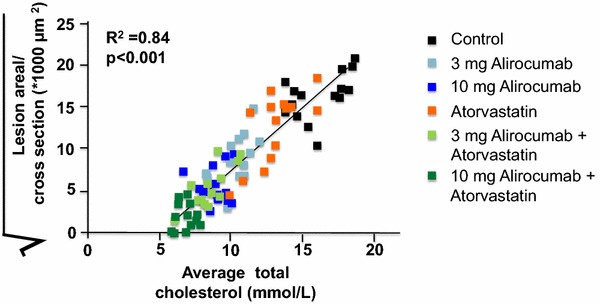
Correlation between average plasma TC and atherosclerotic lesion area in APOE*3Leiden.CETP mice treated with different doses of the PCSK9 inhibitor alone and in combination with atorvastatin. Alirocumab dose-dependently decreased serum cholesterol, reduced atherosclerotic lesion size and improved plaque morphology. These effects were enhanced when atorvastatin was added. (modified from [86])

The GOF-mutation (D374Y) of PCSK9 causes severe hypercholesterolemia and development of profound atherosclerotic lesions in mice [137] and pigs [3]. PCSK9 overexpression increases LDL-C concentration in mice and accelerates the development of atherosclerosis, the latter being absent in LDLR-knockout mice [41]. On the contrary, development of atherosclerosis is slowed down by inactivation of the PCSK9 gene in mice [41]. These data were supported by Kühnast et al. [86] treating mice with increased atherogenesis with different doses of a PCSK9 inhibitor alone and in combination with atorvastatin. Alirocumab alone dose-dependently decreased serum cholesterol, reduced atherosclerotic lesion size and improved plaque morphology. These effects were enhanced when atorvastatin was added [86] (Fig. 4) but beneficial effects require both the presence of the LDLR and apoprotein E [8].

Development of atherosclerosis involves endothelial cell apoptosis and accumulation of foam cells, both of which can be triggered by oxidized LDL-C (oxLDL-C) [154, 165, 174]. Indeed, oxLDL-C increases PCSK9 expression in macrophages [165] and the oxLDL-C induced apoptosis is reduced in human umbilical vein endothelial cells by silencing PCSK9, an effect being related to less caspase 9 and 3 activation [174]. Also cholesterol uptake of THP-1 macrophages and foam cell formation as well as oxLDL-C/NFkB- induced inflammation are attenuated by PCSK9 silencing [165] (for review, see [154]).

**Fig. 5:**
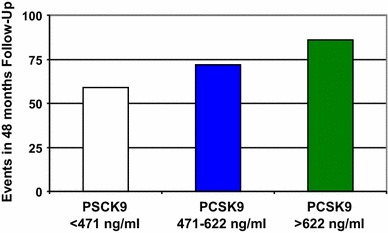
Number of primary outcome events (cardiovascular death and unplanned cardiovascular hospitalization) in 504 consecutive patients with stable coronary artery disease stratified by PCSK9 tertiles after 48 months. Increased PCSK9 serum concentrations correlate with outcomes. (modified from [173])

In line with the above cell and animal experiments, the plasma PCSK9 concentration correlates with the intima-media thickness in patients [96], and GOF mutations of PCSK9 increase not only the LDL-C concentration but also the intima-media thickness over time compared to normal subjects [121]. Plasma PCSK9 concentrations are predictive for 4-5 year major cardiovascular event rate [76] and PCSK9 serum concentrations correlate with cardiovascular risk [95]. In patients with stable coronary artery disease (Fig. 5), higher PCSK9 concentrations were associated with increased cardiovascular events and with female gender, hypertension, statin treatment, C-reactive protein, HbA1c, insulin, total cholesterol and fasting triglycerides, but not with LDL- or HDL-cholesterol. Interestingly, the association of PCSK9 levels with cardiovascular events was reduced after adjustment for fasting triglycerides [173].

**Fig. 6:**
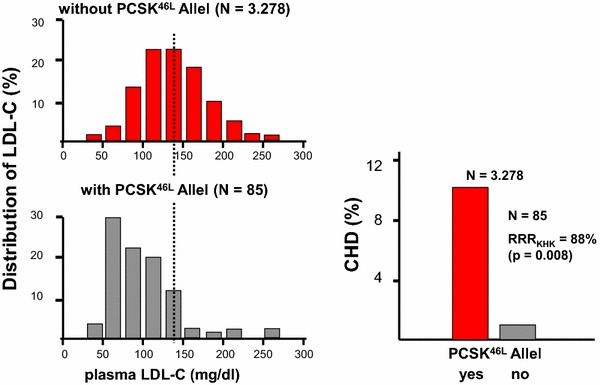
In the Atherosclerosis Risk in Communities study (ARIC), sequence variants of PCSK9 that are associated with reduced plasma LDL-C are associated with reduced risk of coronary heart disease (CHD). The figure depicts the results of *n* = 3363 black subjects: 2.6 % showed nonsense mutations in PCSK9; these mutations were associated with a 28 % reduction in mean LDL-C and a 88 % reduction in the risk of CHD. Of the 9,524 white subjects examined, 3.2 % had a sequence variation in PCSK9 that was associated with a 15 % reduction in LDL-C and a 47 % reduction in the risk of CHD (*P* = 0.003) (modified from [37])

With high-dose statin treatment, however, the predictive value of PCSK9 is lost [76]. Mutations of PCSK9 leading to reduced expression and or function of PCSK9 are associated with a reduced rate of coronary heart disease [37] (Fig. 6), myocardial infarction [63] and overall cardiovascular events [143], an effect being more pronounced in black as compared to white subjects [37].

## Other receptors/channels/enzymes affected by PCSK9

Apart from its binding to LDLR, PCSK9 also interacts with other receptors such as the very low-density lipoprotein receptor (VLDLR) [88, 127], the LDLR-related protein 1 (LRP1) [27], the apoprotein E receptor (ApoER) as well as CD81 on hepatocytes (hepatitis C virus receptor) [89] and CD36 on macrophages (for review, see [154]). Some interactions of PCSK9 with receptors depend on a EGF-A binding domain (VLDLR) [152] or require the catalytic activity of PCSK9 (LRP1). VLDLR and ApoER are also targeted by IDOL (for review, see [76]).

## Lipoprotein (Lp) (a)

Clinical studies show that inhibition of PCSK9 potently lowers Lp(a), which is a strong cardiovascular risk factor [59, 159]. Currently, no drug treatment is available that lowers Lp(a) (with the exeption of nicotinic acid in some countries). Interestingly, PCSK9 inhibition reduces Lp(a) in patients with homozygous FH despite their lack or dysfunction of the LDLR. This effect was observed even in patients that are LDLR negative [159]. Therefore, the question arises whether the regulation of Lp(a) by PCSK9 may be independent of the LDLR. From this perspective, the modulation of VLDLR by PCSK9 appears to be of great interest since Lp(a) clearance by hepatocytes appears to depend on VLDLR expression [73]. Thus, reducing PCSK9 expression or receptor binding activity may mediate the reduction of Lp(a). The underlying molecular mechanism(s) are not fully understood. The following pathways may contribute:

- Reduction of Apo(a) synthesis (in hepatocytes, release in circulation).
- Reduction of ApoB or assembly (at outer hepatocyte surface).
- Enhanced removal of Lp(a) in kidney, liver, peripheral tissues.  Potential additional receptors for Lp(a) such as docking receptors, sorting receptors sortilin, endocytic receptors (syndecan-1 heparan sulfate proteoglycan).
- Intestinal lipoprotein metabolism (please see below).

## Epithelial ENaC

Expression at the cell surface is reduced by PCSK9. PCSK9—independent from its catalytic activity— the proteosomal degradation of ENaC prior to its membrane integration; interestingly and different from other receptor interactions, PCSK9 does not interfere with membrane-bound ENaC [153]. It has been speculated that GOF mutation of PCSK9 will therefore reduce membrane-bound ENaC expression leading to less sodium reabsorption and potentially less hypertension in the long-term. However, the clinical study programs of PCSK9 inhibiting antibodies have not shown signals of increased blood pressure to date.

## Extra-hepatic expression and effects of PCSK9

### Intestinal cholesterol absorption

Although the hepatic effects of PCSK9 appear to be the primary mechanism of action, PCSK9 in intestinal cells may play a very important role for the lipoprotein homeostasis. PCSK9 increases the expression of the apical cholesterol transporter (Niemann Pick C1 like 1, NPC1L1) in intestine epithelial cells [98]. Furthermore, PCSK9 enhances the intracellular expression of the apoprotein B48 (apoB48) [98, 135] and modifies the activities of the HMG-CoA reductase (decreased), the Acyl-CoA-Cholesterol-Transferase (ACAT, decreased) [98] and the microsomal transfer protein (MTP, increased) [98]. It also reduces the expression of the LDLR at the basolateral membrane of intestine epithelial cells [98]. PCSK9 promotes intestinal overproduction of triglyceride-rich apoB lipoproteins [135], and a reduction in PCSK9 leads to a reduced apoB48 expression, less triglycerides being transferred to apoB48 (via MTP inhibition) and prolonged storage and less secretion of triglycerides (via ACAT inhibition) from intestine epithelial cells. As a consequence postprandial triglyceridemia is reduced in PCSK9 knockout mice [95].

Furthermore, the transintestinal cholesterol excretion (TICE)—contributes up to 30 % of fecal neutral sterols excretion—is modulated by PCSK9. TICE is fuelled by apoprotein B containing particles such as LDL and also to a minor extent by HDL. TICE involves the active transporter ATP binding cassette transporter B1 on the apical membrane of enterocytes and the LDLR at its basolateral membrane [94]. PCSK9 through decreasing LDLR expression reduces TICE, as it had no impact in LDLR-knockout mice [94]. These findings may explain the correlation of PCSK9 with triglycerides in the serum [173].

### PCSK9 in the brain

PCSK9 was initially discovered as a protein up-regulated during apoptosis of neurons [150]. PCSK9, that was formerly known as NARC-1 is important for brain development, especially the cerebellum [128]. Here, PCSK9 is thought to interact primarily with VLDLR and ApoER which are coupled to relin signaling and pro-apoptotic signaling pathways [88], although an interaction of PCSK9 with ApoER in brain tissue was questioned more recently [105]. PCSK9 is present in the cerebrospinal fluid at remarkably constant concentrations [33]. Brain tissue PCSK9 expression is increased with cerebral ischemia [88] and in brain tissue with signs of neuronal apoptosis [19], the latter being induced for example by increased oxLDL-C concentrations [176]. A relation to vascular dementia and Alzheimer’s disease is controversial, PCSK9 may be involved in degradation of β-site amyloid precursor protein (APP)-cleaving enzyme 1 and the generation of amyloid β-peptide.

The location of the “loss-of-function” single-nucleotide polymorphism rs11591147 (more commonly called R46L) is depicted in Fig. 1. In the Atherosclerosis Risk in Communities study (ARIC), the R46L mutation was associated with lower LDL-C and a reduced prevalence of peripheral arterial disease as well as a reduced risk of coronary heart disease [17, 37, 57]. Recently, the effects of LDL-C lowering mediated by PCSK9 inhibition on cognitive function have become a matter of debate [http://digitool.library.mcgill.ca/webclient/StreamGate?folder_id=0&dvs=1420384772483~463]. Jukema et al. therefore assessed the PCSK9 R46Lmutation within 5,777 participants of the PROspective Study of Pravastatin in the Elderly at Risk (PROSPER) [129]. R46L was associated with 10–16 % lower LDL-C levels, but was not associated with cognitive performance, daily activities, or non-cardiovascular clinical events. The authors conclude “that lower cholesterol levels due to genetic variation in the PCSK9 gene are not associated with cognitive performance, functional status, or non-cardiovascular clinical events” [129].

### PCSK9 in pancreatic ß-cells

PCSK9 and LDLR are also expressed in ß-cells. PCSK9-knockout mice carry more LDLR and less insulin in the pancreas, leading to hyperglycemia and glucose intolerance. ß-cell islets of PCSK9-knockout mice inhibit signs of inflammation and apoptosis [112]. This phenotype is modulated by gender and age [111]. Glucose tolerance is one of the parameters that will be carefully monitored in the outcome trials with PCSK9 inhibitors, so far the phase II data do not suggest the presence of this potential off-target effect in humans.

### PCSK9 in adipose tissue

PCSK9-knockout mice have more visceral adipose tissue. Individual adipocytes are hypertrophied, most likely as a result of increased expression of VLDLR [141].

### PCSK9 and innate immune response

Pathogen-associated lipids such as lipopolysaccharide (LPS) activate innate immune receptors inducing an inflammatory response, e.g. during sepsis. Mammalian lipid transfer proteins bind pathogen lipids. Interstingly, PCSK9 inhibited LPS uptake in human liver cells [171]. Inhibition of PCSK9 improved survival and inflammation in murine sepsis. The PCSK9 effect was abrogated in LDL receptor (LDLR) knockout mice. These data were confirmed in humans with PCSK9 loss-of-function genetic variants and in humans who are homozygous for an LDLR variant that is resistant to PCSK9. These data suggest that inhibition of PCSK9 mediates pathogen lipid clearance via the LDLR regulating systemic inflammatory response.

## Therapeutic strategies to inhibit PCSK9

### Anti-PCSK9 antibody treatment

Fully human antibodies directed against PCSK9 have been developed and are used in clinical trials (for detailed review, see [78]). These antibodies dose-dependently reduce plasma LDL-C and also lower the plasma Lp(a) concentration. Results of published trials are given in Table 2. However, new strategies to modify the PCSK9-LDLR interaction are in development (Table 3).

**Table 2:** Clinical results of PCSK9 antibody treatment

Study name	Substance	Patients’ characteristics	Patient number	Analysis done at	Co-treatment	LDL reduction	Other lipid parameters	Reference
First author								
Odyssey alternative	Alirocumab (Ali)	Statin intolerant	Ali: 126 Ezetimibe	24 weeks	Ali	Ali: 52 %	Ali: Lp(a)-27 %	AHA2014^a^
					Eze	Eze: 17 %	Ali: ApoB-43 %	
			(Eze): 125				Eze:Lp(a)-10 %	
							Eze:ApoB-14 %	
Odyssey Combo 2	Alirocumab	High CV risk	Ali: 479	52 weeks	Atorvastatin (Ator)	Ali + Ator: 50 %	Not presented	ESC2014^b^ [38]
						Ali + Eze: 18 %		
			Eze: 241					
Odyssey FHI + FHII	Alirocumab	HeFH	735	52 weeks	Statins and/or Eze	FH I: 49 %	Not presented	ESC2014^b^
						FH II: 51 %		
Odyssey long-term	Alirocumab	HeFH or high CV risk	2,421	52 weeks	Ator and/or Eze	Ali: 56 %	Ali: Lp(a)-26 %	ESC2014^c^
							Ali: ApoB-54 %	
McKenney	Alirocumab	LDL-C > 100	183	12 weeks	Statins	Ali: 40–70 %, dose- and administration- dependent	Not presented	[113]
Roth	Alirocumab	LDL-C >100, <190 and moderate CV risk	103	24 weeks	Eze	Eze: 17 %	Not presented	[140]
						Ali: 54 %		
Roth	Alirocumab	LDL-C <100 on atorvastatin (10 mg)	92	8 weeks	Ator 80 mg vs. Ali + Ator 10 mg	Ato: 17 %	Not presented	[139]
						Ali: 72 %		
Stein	Alirocumab	HeFH	77	12 weeks	Statins or Eze	Ali: 29–68 %; dose-administration dependent	Not presented	[157]
Stein	Alirocumab	HeFH; LDL-C <100	51	12 weeks	Statins	Ali: 39–61 %; dose-administration dependent	Not presented	[160]
Meta analysis	Alirocumab	Some of the above study patients with hypercholesterolemia	186	12 weeks	Standard of care	Not presented	Lp(a): −30 %	[59]
Mendel-1	Evolocumab (Evo)	LDL-C <190	406	12 weeks	Eze	Evo: 37–52 %	Not presented	[83]
						Eze: 27–34 %		
Mendel-2	Evolocumab	LDL-C <190; Framingham score <10 %	614	12 weeks	Eze	Evo: 55–57 %	Not presented	[82]
						Eze: 38–10 %		
GAUSS-1	Evolocumab	Statin intolerant	157	12 weeks	Eze	Evo: 26–47 %	Not presented	[164]
GAUSS-2	Evolocumab	Hypercholesterolemia Statin-intolerant	307	12 weeks	Evo	Evo: 53–56 %	Not presented	[35, 161]
					Eze	Eze: 37–39 %		
Laplace-TIMI	Evolocumab	LDL-C >85 mg on statins and/or Eze	631	12 weeks	Statins	Evo: 42–62 %; dose-and administration- dependent	Evo: Lp(a)—18–23 %	[43, 60]
Laplace-TIMI	Evolocumab	High CV risk	282	12 weeks	Statins		Not presented	[42]
LAPLACE 2	Evolocumab	Statins low dose LDL >115	2,067	12 weeks	Statins	Evo: 63–75 %; dose-administration dependent	Not presented	[136]
		high-dose LDL >90						
Rutherford	Evolocumab	HeFH	167	12 weeks	Statins and/or ezetimibe	Evo: 43–55 %	Not presented	[131]
Rutherford 2	Evolocumab	HeFH	331	12 weeks	Statins	Evo: 59–61 %; dose-administration dependent	Not presented	[134]
Osler	Evolocumab	Mendel; Laplace; Gauss and Rutherford patients	1,104	52 weeks	Standard of care	Evo: on average 50 %	Not presented	[81]
YUKAWA	Evolocumab	High CV risk	310	12 weeks	Statins and/or ezetimibe	Evo: 52–68 %	Not presented	[69]
Dias	Evolocumab	META-analysis	113	12–16 weeks	Statins	Evo: 64–81 %; dose-administration dependent	Not presented	[46]
Blom	Evolocumab	Hypercholesterolemia	901	52 weeks	Diet (D), Eze, Ator	Evo + D: 56 %	Reduced ApoB, nonHDL-C, Lp(a), triglycerides	[20]
						Evo + Ator10 mg: 61.6 %;		
						Evo + Ator 80 mg: 56.8 %		
						Evo + Ator + Eze: 48.5 %		
Stein	Evolocumab	Homozygous FH	6	12 weeks	Standard of care	Evo: 19–26 %	Not presented	[159]
Tesla	Evolocumab	Homozygous FH	49	12 weeks	Standard of care	Evo: 31 %	Not presented	[133]
Metaanalysis	Evolocumab	Some of the above study patients with hypercholesterolemia	1,359	12 weeks	Standard of care	Evo: 40–59 %	Evo: Lp(a) 25–30 %	[132, 158]

*He* heterozygous, *FH* familiar hypercholesterolemia, *CV* cardiovascular risk, *LDL-C* LDL-cholesterol presented in mg/dl

^a^Patrick M Moriarty; http://my.americanheart.org/idc/groups/ahamah-public/@wcm/@sop/@scon/documents/downloadable/ucm_469684.pdf

^b^Christopher Paul Cannon, Michel Farnier: http://www.escardio.org/congresses/esc-2014/congress-reports/Pages/707-3-Hotline3-ODYSSEY-COMBO-FH.aspx#.VHrMIsk2xJQ

^c^Jennifer Robinson; http://www.escardio.org/congresses/esc-2014/congress-reports/Pages/707-4-Hotline3-ODYSSEY-Long-term.aspx#.VHrMOsk2xJQ

**Table 3:** PCSK9 inhibitors in development

Type	Compound	Company	Phase	Comments
mAb	Evolocumab AMG145	Amgen	3 PROFICIO	
	Alirocumab REGN7272/SAR236553	Sanofi/regeneron	3 ODYSSEY	
	Bococizumab RN-316;PF-04950615	Pfizer/rinat	3 SPIRE	
	RG7652	Roche/genentech	2	On hold
	LY3015014	Eli Lilly	2	
	LGT209	Novartis	2	
Adnectin	Ad. BMS-962476	BMS-Adnexus	2	
siRNA	ALN-PCS	Alnylam Pharmaceuticals	1 (IV); preclinical (SC)	Cationic lipidoid formula
Small molecule	–	Shifa Biomedical Corp	Preclinical	Preparation for Phase 1
Mimetic peptide	EGF-A peptide	Merck & Co.	Preclinical	
	Prodomain and C-terminal domain interaction disruption	School of Medicine, University of South Carolina, USA	Preclinical	

### Partial antibodies/fragment antigen binding

Antibodies to PCSK9, binding to epitopes adjacent to the ones required for LDLR binding, increase LDLR expression in hepatocytes. This antibody administered to mice results in a significant reduction in plasma LDL-C concentration which is not seen in LDLR-knockout mice. The effect of antibody treatment on LDL-C is even more pronounced and prolonged in monkeys [30]. Similar results are obtained with antibodies covering the catalytic domain of PCSK9 otherwise binding the EGF-A domain of the LDLR [119, 180]; such treatment reduces the free plasma PCSK9 and LDL-C concentrations in rhesus monkeys [146]. Similarly, antibodies against the C-terminal domain lower LDL-C in cynomolgus monkeys [145].

Similar to antibodies, molecular scaffolds binding to PCSK9 close to its LDLR binding epitopes reduce the free PCSK9 and subsequently the LDL-C concentration in cynomolgus monkeys [118]. Such a molecular scaffold is adnectin (11 kDa), which is derived from human fibronectin.

Apart from the interaction with secreted PCSK9, interference can occur at the gene or mRNA level. Induction of loss-of-function mutations in mice, using clustered regularly interspaced short palindromic repeats (CRISPR-Cas9) genome editing, results in reduced plasma PCSK9 and LDL-C concentrations [155]. RNA interfering (RNAi) drugs attenuate PCSK9 synthesis and decrease plasma PCSK9 and LDL-C concentrations in rodents [58], primates [58] and healthy volunteers [56]. Locked nucleic acid (LNA) antisense oligonucleotide silences PCSK9 in vitro (hepatocytes) and in vivo (mice) subsequently reducing plasma PCSK9 and LDL-C concentrations [65]. Again, similar results are obtained in non-human primates where a single injection of LNA decreases LDL-C concentration for more than 4 weeks [103].

PCSK9 has both intracellular and extracellular functions which differ related to the cell type and organ. PCSK9 affects both receptor expression and intracellular enzyme function. Thus, antibodies directed against the secreted form of PCSK9—acting mainly extracellularly—will most likely lead to different results when compared to approaches resulting to LOF-mutation or gene knockout of PCSK9, both interfering PCSK9 intracellular and extracellular function. Epidemiological findings related to GOF- or LOF- mutations of PCSK9 may therefore not easily be transferred to recent antibody approaches.

In conclusion, effects of PCSK9 inhibitors in addition to the regulation of the LDLR expression clearly exist and may be beneficial. In the large on-going phase 3 programs using PCSK9 inhibitors, to date no “loss of safety”- signal has been observed. The primary effect of PSCK9 inhibition appears to be the up-regulation of hepatic LDLR but further basic science and clinical research will advance our understanding of how PCSK9 inhibition interferes with lipoprotein metabolism and improve cardiovascular outcome.
